# Discordant Cavitary Changes: A Radiological Dilemma in Invasive Mucinous Adenocarcinoma: A Case Report

**DOI:** 10.1002/rcr2.70791

**Published:** 2026-10-19

**Authors:** Akina Nigi, Noritaka Miya, Keisuke Iwamoto, Hidetoshi Itani, Shigeto Kondou

**Affiliations:** ^1^ Department of Respiratory Medicine Japanese Red Cross Ise Hospital Ise Mie Japan

**Keywords:** bevacizumab, cavitation, invasive mucinous adenocarcinoma, lung cancer, radiological response

## Abstract

Treatment‐related cavitary transformation in lung cancer may complicate radiological response assessment. We report a man in his 70s with stage IVA invasive mucinous adenocarcinoma treated with atezolizumab, bevacizumab, carboplatin and paclitaxel. After two cycles, CT showed enlargement of cavitary components despite regression of solid tumour components, making response assessment difficult. After two additional cycles, the pulmonary lesions showed little change, whereas progression of the Th4 bone metastasis led to an overall assessment of progressive disease and a change to docetaxel plus ramucirumab. Thereafter, the cavities further enlarged while the cavity walls progressively thinned and the solid components appeared to regress. This case illustrates that marked multifocal cavitary transformation may produce discordant morphological changes, limiting response assessment based on imaging alone. Serum CEA and SLX levels remained within the normal ranges at diagnosis and throughout treatment.

## Graphical abstract

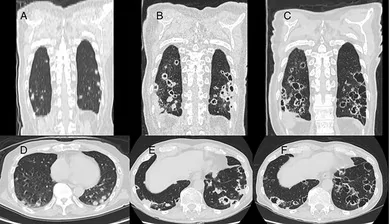

## 1 Introduction

The Response Evaluation Criteria in Solid Tumors (RECIST) are widely used to assess treatment response in solid malignancies [1]. However, in pulmonary lesions showing treatment‐related cavitary transformation, changes in the external lesion diameter may be discordant with changes in the residual solid components, making response assessment difficult. We report a case of invasive mucinous adenocarcinoma in which cavitary lesions enlarged despite progressive thinning of the cavity walls and regression of the solid components, resulting in difficulty in radiological response assessment.

## 2 Case Report

A man in his 70s was referred to our hospital on December 6, X, because of multiple pulmonary nodules (Figure 1A). He had a smoking history of 10 cigarettes per day for approximately 10 years in his 20s–30s. He was diagnosed with invasive mucinous adenocarcinoma of the lung (Figure 2A–D), staged as cT4N1M1b, stage IVA. No actionable driver alterations were detected, and programmed death‐ligand 1 expression was 15%. Palliative radiotherapy, 35 Gy in 5 fractions, was administered for a Th4 vertebral metastasis.

**FIGURE 1:**
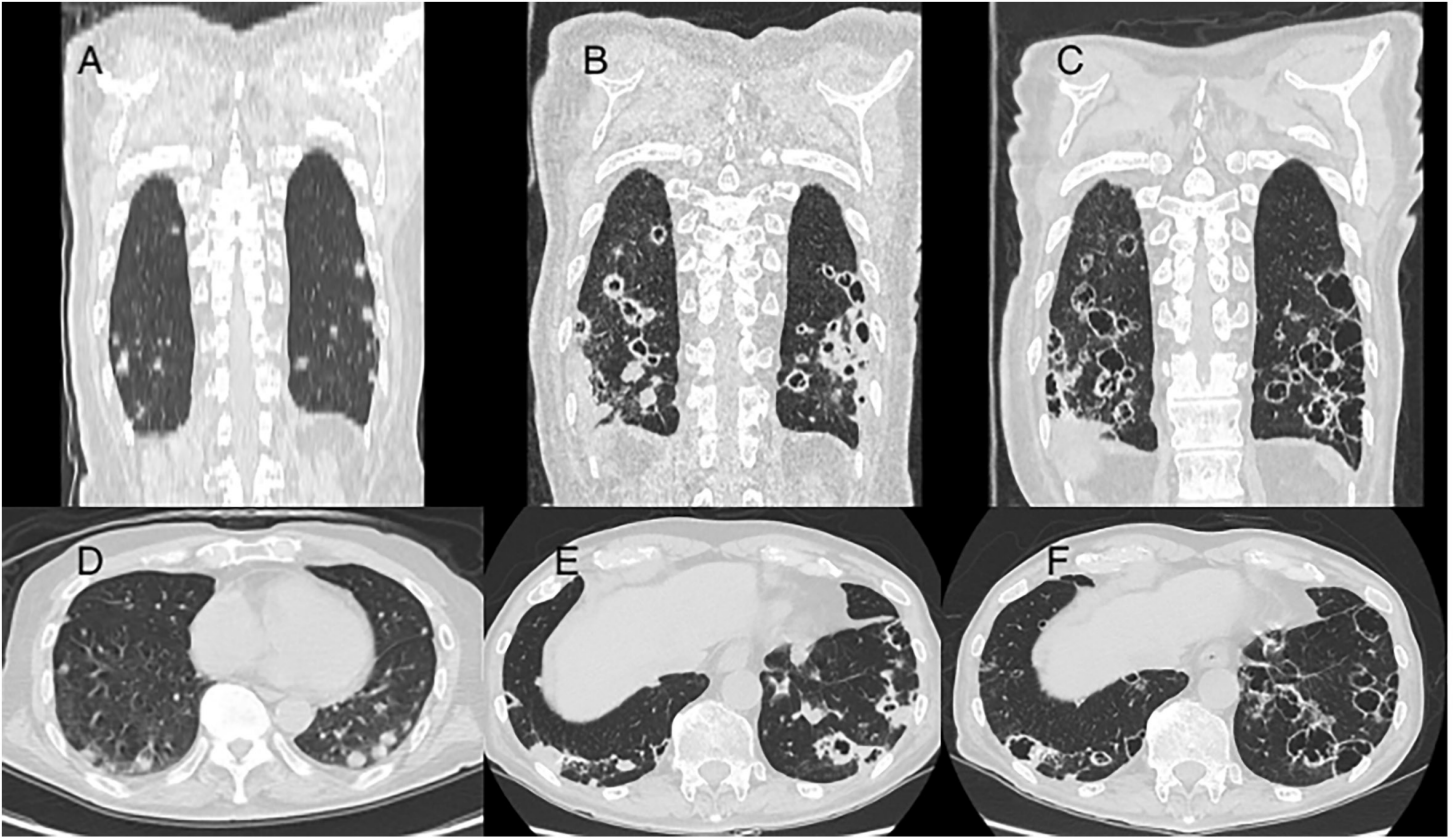
Serial coronal and matched axial CT images demonstrating discordant cavitary changes during treatment. (A, D) Baseline CT showing multiple bilateral pulmonary lesions with solid components. (B, E) After two cycles of atezolizumab, bevacizumab, carboplatin and paclitaxel, marked cavitary transformation developed. (C, F) During docetaxel plus ramucirumab therapy, multiple cavitary lesions further enlarged despite progressive thinning of the cavity walls.

**FIGURE 2:**
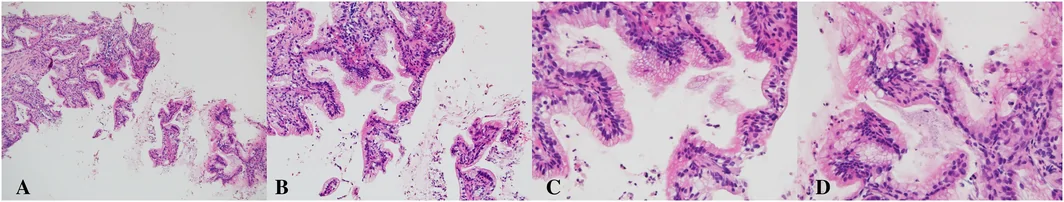
Histopathological findings of invasive mucinous adenocarcinoma (haematoxylin and eosin staining). (A, B) Low‐power views showing papillary and lepidic proliferation of mucin‐producing tumour cells. (C, D) High‐power views demonstrating mucin‐producing columnar tumour cells with abundant intracellular mucin, consistent with invasive mucinous adenocarcinoma.

Systemic chemotherapy with atezolizumab, bevacizumab, carboplatin and paclitaxel was initiated on January 26, X + 1. Follow‐up CT after two cycles showed regression of the solid components of the tumours, whereas enlargement of cavitary components was observed (Figure 1B). Although a CT scan performed after two additional courses of treatment showed no significant changes in the lung fields, the progression of Th4 bone metastasis led to the conclusion that the tumour had progressed. The overall disease was therefore judged as progressive, and treatment was changed to docetaxel plus ramucirumab.

After three cycles of docetaxel plus ramucirumab, CT demonstrated further enlargement of multiple cavitary lesions with progressive thinning of the cavity walls (Figure 1C). At that time, radiological response assessment was difficult, because the findings were discordant: the cavitary lesions enlarged, whereas the solid components appeared to regress. One additional cycle was administered, but chemotherapy was discontinued because of declining activities of daily living. The patient subsequently received no further chemotherapy and died on February 6, X + 2, approximately 4 months after the final chemotherapy. Serum CEA and SLX levels were within the normal ranges throughout treatment. Throughout the course of treatment, there were no instances of haemoptysis or the need for oxygen therapy.

## 3 Discussion

Cavitary lesions in lung cancer are commonly observed in squamous cell carcinoma, whereas cavitation is relatively uncommon in adenocarcinoma [2, 3]. Cavitary change may also occur as a treatment‐related radiological finding, although it is infrequent. Proposed mechanisms include central tumour necrosis, ischaemia, communication with the bronchial tree, treatment‐related reduction in viable tumour cells and alterations in tumour blood flow induced by antiangiogenic agents.

Tumour cavitation has been reported during antiangiogenic therapy for non‐small cell lung cancer. Inhibition of the vascular endothelial growth factor pathway may cause vascular regression, intratumoural ischaemia and necrosis, thereby promoting cavitary transformation [4]. In the present case, marked cavitary changes developed during treatment with atezolizumab, bevacizumab, carboplatin and paclitaxel and progressed further during docetaxel plus ramucirumab therapy. Thus, antiangiogenic therapy may have contributed to the cavitary transformation, although the precise mechanism remains uncertain.

Radiological response assessment was particularly difficult in this case. After ABCP therapy, enlargement of the cavitary components increased the apparent overall dimensions of the lesions, despite regression of the solid components. On the CT scan taken after two cycles of ABCP, tumour progression could not be determined based on the images alone; therefore, a CT scan was performed after two additional courses, and at that point, worsening of bone metastases in Th4 was observed. Therefore, the disease was judged as progressive. A limited‐field CT obtained on January 24, X + 1, 2 days before initiation of ABCP, primarily for evaluation of the Th4 vertebral metastatsis, showed no cavitary changes in the included pulmonary field. However, because the entire lungs were not covered, interval changes in non‐imaged pulmonary lesions could not be completely excluded.

In contrast, during docetaxel plus ramucirumab therapy, the cavities enlarged while their walls became progressively thinner and the solid components appeared to regress.

These discordant findings raised the possibility of treatment effect; however, the overall response could not be determined with sufficient objective evidence.

As highlighted by Zhang et al. [5], an optimal method for evaluating cavitary lesions during lung cancer treatment has not yet been established. Consequently, deciding whether to continue or modify treatment can be particularly challenging in such cases. Serum CEA and SLX levels were within the normal ranges at diagnosis and were therefore not useful for monitoring treatment response. This case illustrates that marked multifocal cavitary transformation may produce discordant morphological changes that complicated radiological assessment. Response assessment should therefore incorporate cavity‐wall thickness, residual solid components, overall tumour distribution and the clinical course, rather than relying solely on the external diameter of the lesions. RECIST remains a practical and useful standard for response assessment in most patients. However, in uncommon cases showing marked cavitary transformation, particularly when cavity enlargement is accompanied by progressive thinning of the cavity walls, conventional size‐based assessment may be difficult. In such situations, clinicians may be required to make treatment decisions despite persistent uncertainty regarding treatment response.

## Author Contributions

**Akina Nigi:** conceptualization, investigation, formal analysis, writing – review and editing, writing – original draft. **Noritaka Miya:** writing – review and editing. **Keisuke Iwamoto:** writing – review and editing. **Hidetoshi Itani:** writing – review and editing. **Shigeto Kondou:** supervision, writing – review and editing.

## Consent

The authors declare that written informed consent was obtained for the publication of this manuscript and accompanying images using the form provided by the Journal.

## Conflicts of Interest

The authors declare no conflicts of interest.

## Data Availability

The data that support the findings of this study are available on request from the corresponding author. The data are not publicly available due to privacy or ethical restrictions.
